# Stabilization of UCA1 by N6-methyladenosine RNA methylation modification promotes colorectal cancer progression

**DOI:** 10.1186/s12935-021-02288-x

**Published:** 2021-11-22

**Authors:** Rong-Zhang He, Jing Jiang, Xinglin Hu, Ming Lei, Jia Li, Weihao Luo, Lili Duan, Zheng Hu, Yin-Yuan Mo, Di-Xian Luo, Wan-Xin Peng

**Affiliations:** 1 Translational Medicine Institute, National and Local Joint Engineering Laboratory for High-Through Molecular Diagnosis Technology, The First People’s Hospital of Chenzhou, The First Affiliated Hospital of Xiangnan University, Chenzhou, 423000 China; 2 Department of Clinical Pharmacology, Xiangya Hospital, Central South University, Changsha, 410078 Hunan China; 3 Center of Medical Laboratory, The First People’s Hospital of Chenzhou, University of South China, Chenzhou, 423000 China; 4 Department of Dermatology, Affiliated the First People’s Hospital of Chenzhou of University of South China, Chenzhou, 423000 China; 5 Department of Clinical Laboratory, The First People’s Hospital of Changde City, Changde, 415003 China; 6 Cancer Institute, University of Mississippi Medical Center, Jackson, MS USA; 7 Department of Laboratory Medicine, Huazhong University of Science and Technology Union Shenzhen Hospital (Nanshan Hospital), Guangdong, 518000 China; 8 Present Address: National Clinical Research Center for Child Health, National Children’s Regional Medical Center, the Children’s Hospital, Zhejiang University School of Medicine, No. 3333, Binsheng Road, Hangzhou, 310052 China

**Keywords:** CRC, UCA1, m6A modification, IGF2BP2

## Abstract

**Background:**

UCA1 is frequently upregulated in a variety of cancers, including CRC, and it can play an oncogenic role by various mechanisms. However, how UCA1 is regulated in cancer is largely unknown. In this study, we aimed to determine whether RNA methylation at N6-methyladenosine (m6A) can impact UCA1 expression in colorectal cancer (CRC).

**Methods:**

qRT-PCR was performed to detect the level of UCA1 and IGF2BP2 in CRC samples. CRISPR/Cas9 was employed to knockout (KO) UCA1, METTL3 and WTAP in DLD-1 and HCT-116 cells, while rescue experiments were carried out to re-express METTL3 and WTAP in KO cells. Immunoprecipitation using m6A antibody was performed to determine the m6A modification of UCA1. In vivo pulldown assays using S1m tagging combined with site-direct mutagenesis was carried out to confirm the recognition of m6A-modified UCA1 by IGF2BP2. Cell viability was measured by MTT and colony formation assays. The expression of UCA1 and IGF2BP2 in TCGA CRC database was obtained from GEPIA (http://gepia.cancer-pku.cn).

**Results:**

Our results revealed that IGF2BP2 serves as a reader for m6A modified UCA1 and that adenosine at 1038 of UCA1 is critical to the recognition by IGF2BP2. Importantly, we showed that m6A writers, METTL3 and WTAP positively regulate UCA1 expression. Mechanically, IGF2BP2 increases the stability of m6A-modified UCA1. Clinically, IGF2BP2 is upregulated in CRC tissues compared with normal tissues.

**Conclusion:**

These results suggest that m6A modification is an important factor contributing to upregulation of UCA1 in CRC tissues.

**Supplementary Information:**

The online version contains supplementary material available at 10.1186/s12935-021-02288-x.

## Introduction

Colorectal cancer (CRC) is the third most commonly diagnosed cancer, and is the second leading cause of cancer-related death worldwide [1]. In China, CRC is one of the top five diagnosed cancers and causes of cancer-related deaths [2]. Despite declines in incidence over the past decade owing to adoption of effective screening programs, approximately 50% of patients show metastasis at the time of diagnosis [3]. Moreover, the incidence of early-onset CRC has increased at an alarming rate [4, 5]. Therefore, there is a pressing need to elucidate the detailed mechanisms of CRC carcinogenesis to develop effective strategies against CRC.

Long non-coding RNA (lncRNA) is a transcript with a length > 200 nucleotides, lacking the coding capacity. Evidence accumulated over the past decade indicates that the lncRNAs are frequently dysregulated in cancer and they can play key roles in tumorigenesis. In particular, CRC represents one of the tumors showing the most relevant dysregulation of lncRNA functions [6, 7]. Numerous lncRNAs have been identified to be differentially expressed in CRC, participating in many aspects of CRC development, such as proliferation, metastasis, anti-apoptosis and energy metabolism [7–11]. Urothelial carcinoma associated 1 (UCA1) is one of the most well-known lncRNAs and is highly expressed in CRC and tightly associated with the development and progression of CRC [12–14]. Recent studies further show that the circulating UCA1 from blood may serve as a new diagnostic biomarker in patients with CRC [15, 16].

Although the crucial role of UCA1 in CRC development has been well characterized, the mechanism underlying the upregulation of UCA1 in CRC is still largely unknown. In this study, we demonstrated that m6A modification of UCA1 enhances its RNA stability, resulting in the high level of UCA1 in CRC. We further showed that knockout of m6A writer METTL3 or WTAP decreases UCA1 RNA level in CRC cells, whereas rescue experiment (i.e., re-expression of METTL3 or WTAP in the corresponding KO cells) restores the expression level of UCA1. In vivo RNA pulldown assay indicated that UCA1 is recognized by IGF2BP2 on the m6A site in CRC cells. Silencing of IGF2BP2 impairs the RNA stability of UCA1, highlighting the importance of m6A modification in regulation of UCA1 expression in CRC.

## Materials and methods

### Reagents

WTAP (56501) and METTL3 (96391) were purchased from Cell Signaling Technology (Danvers, MA), YTHDF1 (17479-1-AP), YTHDF2 (24744-1-AP), YTHDF3 (25537-1-AP), IGF2BP1 (22803-1-A), IGF2BP2 (11601-1-AP), IGF2BP3 (14642-1-AP) were from Proteintech (Chicago, IL, USA). Secondary antibodies conjugated with IRDye 680 were purchased from LICOR Biosciences (Lincoln, NE, USA). Anti-N6-methyladenosine antibody (17-3-4-1) were purchased from MilliporeSigma (St. Louis, MO, USA). PCR primers were purchased from IDT (Coralville, IA, USA).

### Patients and samples

We collected 46 patients with CRC diagnosed in the First People’s Hospital of Chenzhou City between 2014 and 2016. This study was approved by the Ethics Committee of the First People’s Hospital of Chenzhou. All procedures were implemented according to the guidelines of the Declaration of Helsinki. All patients provided written informed consent. Fresh colorectal neoplasms and matching normal tissues (located > 2 cm away from the tumor boundary) were stored in liquid nitrogen until RNA isolation.

### Cell culture

HCT116, DLD-1, 293 T were purchased from ATCC (Manassas, VA). HCT116 and DLD-1 were cultured with RPMI 1640 from Sigma supplemented with 10% fetal bovine serum (FBS, Gibco, Sigma). 293 T cells were grown in DMEM with 10% FBS. All media were supplemented with 2mM L-glutamine, 100 U/mL penicillin, and 100 mg/mL streptomycin (Lonza, Walkersville, MD). Cells were incubated at 37 ℃ and supplemented with 5% CO2 in the cell incubator.

### Transfection

Cells were transfected with plasmid or siRNAs using Lipofectamine 2000 transfection reagent (Life Technologies) according to the manufacturer’s instruction. The siRNA sequences targeting IGF2BP2 were designed and synthesized by Genepharma Company (Shanghai, China), and they were listed in Additional file 1: Table S1.

### Lentivirus infection

Lentivirus was packaged in 293 T cells using the third generation of packaging system. Virus was collected 48 h after transfection. Lentivirus infection was carried out 6-well plates by mixing 500 μl virus supernatant and 500 μl medium containing 8 μg polybrene.

### Quantitative RT-PCR

Total RNA was isolated from cells using Trizol reagent (Invitrogen), and then cDNA was synthesized using GoScript™ RT System (Promega, USA). Quantitative RT-PCR (qRT-PCR) was performed using a standard SYBR Green method (Bio-Rad, Hercules, CA, USA). Actin was used as an internal control. The primers were listed in Additional file 1: Table S1.

### Cell proliferation assay

Cell proliferation was performed using Cell Counting Kit-8 (CCK-8) Kit (Dojindo, Kyushu, Japan). According to the manufacturer’s instruction, as previously described [17]. Briefly, 3 × 10^3^ /well HCT-116 or DLD-1 cells were seeded into a 96-well flat-bottomed plate. Cell numbers were evaluated for next 3 days. Ten microliters of CCK-8 reagent was added to each well, after which the plate was incubated at 37 °C for 2 h. Subsequently, the absorbance at 450 nm was measured in each well by using a spectrophotometer (Molecular Devices, CA, USA). All assays were performed in triplicate.

### Colony formation assay

For the colony formation assay, 500 cells/well in the growth phase were seeded in six-well plates. The cells were cultured in cell incubator at 37 °C in 5% CO_2_ for 10–15 days. Then the cells were fixed with 4% paraformaldehyde and stained with crystal violet dye. The number of macroscopic colonies was counted, and images were obtained of the representative colonies.

### Western blot

Cells were harvested and proteins were extracted and quantified as previously described [18]. Protein samples were subjected to SDS–polyacrylamide gel electrophoresis (SDS-PAGE) and then transferred to polyvinylidene difluoride membranes (PVDF). The membranes were incubated with primary antibodies followed by h a secondary antibody labeled with either IRDye 680. Finally, signal intensity was determined using the Odyssey Infrared Imaging System (LICOR Biosciences, Lincoln, NE, USA).

### Plasmid construction

WTAP (#53741) and METTL3 (#53739) expression vectors for rescue experiments were obtained from Addgene [19]. S1m expression vector carried four copies of modified S1 (S1m) sequences; these four copies were sequentially cloned into pCDH-CAG-EF1- copGFP-T2A-Pu (derived from pCDH-MSCV-copGFPT2A-Pu) at EcoR1 and BamH1 sites, resulting in S1m tagging vector, pCDH-CAG-4xS1m-EF1-copGFP-T2A-Pu. UCA1 was first amplified using primers UCA1-S1m-R1-5.1 and UCA1-S1m-BamH1-3.1 and then cloned into this S1m expression vector at EcoR I and BamH I sites. Mutation of UCA1 at a putative m6A site was made by two overlapped PCR products using primers UCA1-S1m-R1-5.1 and UCA1-mut-3.1; UCA1-mut-5.1 and UCA1-S1m-BamH1-3.1, and then cloned into S1m vector at EcoR I and BamH I sites. PCR reactions for cloning purpose used Phusion enzyme (ThermoFisher Scientific). All PCR products were verified by DNA sequencing.

### KO experiments

CRISPR/Cas9 system [20] was used to knock out WTAP, METTL3 and UCA1, respectively, using a dual gRNA approach as described previously [17] using the optimized scaffold [21] to generate LCV2-m. Sequences for each pair of gRNAs were listed in Additional file 1: Table S1. The lentiviral vector carrying Cas9 and dual gRNAs were introduced into HCT116 or DLD1 cells by infection. Three days after infection, the cells were subject to puromycin selection. Two weeks later, individual colonies were manually collected and expanded in 12-well plates. The potential clones were further verified by qRT-PCR or Western blot.

### RNA immunoprecipitation (RIP)

We used m6A antibody to pull down m6A modified RNA, and IGF2BP2 antibody to pull down RNA. Total RNA was extracted from a 10 cm dish and dissolved in 50 μl RNase-free water. We used 5 μl as RNA input and the remaining RNA was added to 500 μl pulldown lysis buffer containing RNase inhibitor. In the case m6A pulldown, the total RNA was first precleaned with 1 µl mouse IgG and then the IgG was removed by protein A/G beads, followed by incubation with control IgG or primary antibody overnight at 4 °C. Finally, precipitated RNA was isolated with Trizol and the RNA level of UCA1 was determined by qRT-PCR.

### In vivo S1m precipitation

The procedure of in vivo pulldown of UCA1 using S1m-tagged system was similar to what had been previously described [17]. The HCT116 cells in a 10 cm dish were transfected with S1m vector or S1m-UCA1. The cells were harvested 48 h after transfection and then lysed in 1 ml lysis buffer containing protein inhibitors and RNase inhibitor. After centrifugation for 20 min at 4 °C, the supernatant was collected and incubated with streptavidin beads for 30 min at 4 °C. After spin to remove the streptavidin beads, the precleaned cell lysate was transferred to a new tube, followed by incubation with a new batch of streptavidin beads for 4 h at 4 °C, then washing 5 times with ice-cold PBS. Finally, the pellet was dissolved in 30 μl 2× SDS sample buffer, followed by SDS-PAGE and Western blot with YTHDF1, YTHDF2, YTHDF3, IGF2BP1, IGF2BP2 and IGF2BP3 antibody, respectively.

### Animal experiment

BALB/c nude mice (4–6 weeks old) were purchased from Hunan SLAC Jingda Experimental Animal Company (Changsha, China). All animal experiments were approved by the Ethical Committee of the First People’s Hospital of Chenzhou City, University of South China. Six mice in each group were subcutaneously injected with 5.0 × 10^6^ stable HCT-116 UCA1-KD cells or control cells in 0.2 mL PBS with 50% matrigel as described previously [22]. Five weeks after the injection, the nude mice were sacrificed by cervical dislocation after overdose Isoflurane anesthetization. The dorsal tumors were excised and weighed after they were photographed.

### Statistical analysis

All statistical analyses were performed using the GraphPad Prism program. The continuous variables are summarized as mean and standard deviation (SD) unless stated. One-way analysis of variance (ANOVA), paired t test and unpaired t test were used for statistical comparison. All P values were two-sided and P values < 0.05 were considered as significant.

## Results

### Upregulation of UCA1 in CRC tissues

**Fig. 1:**
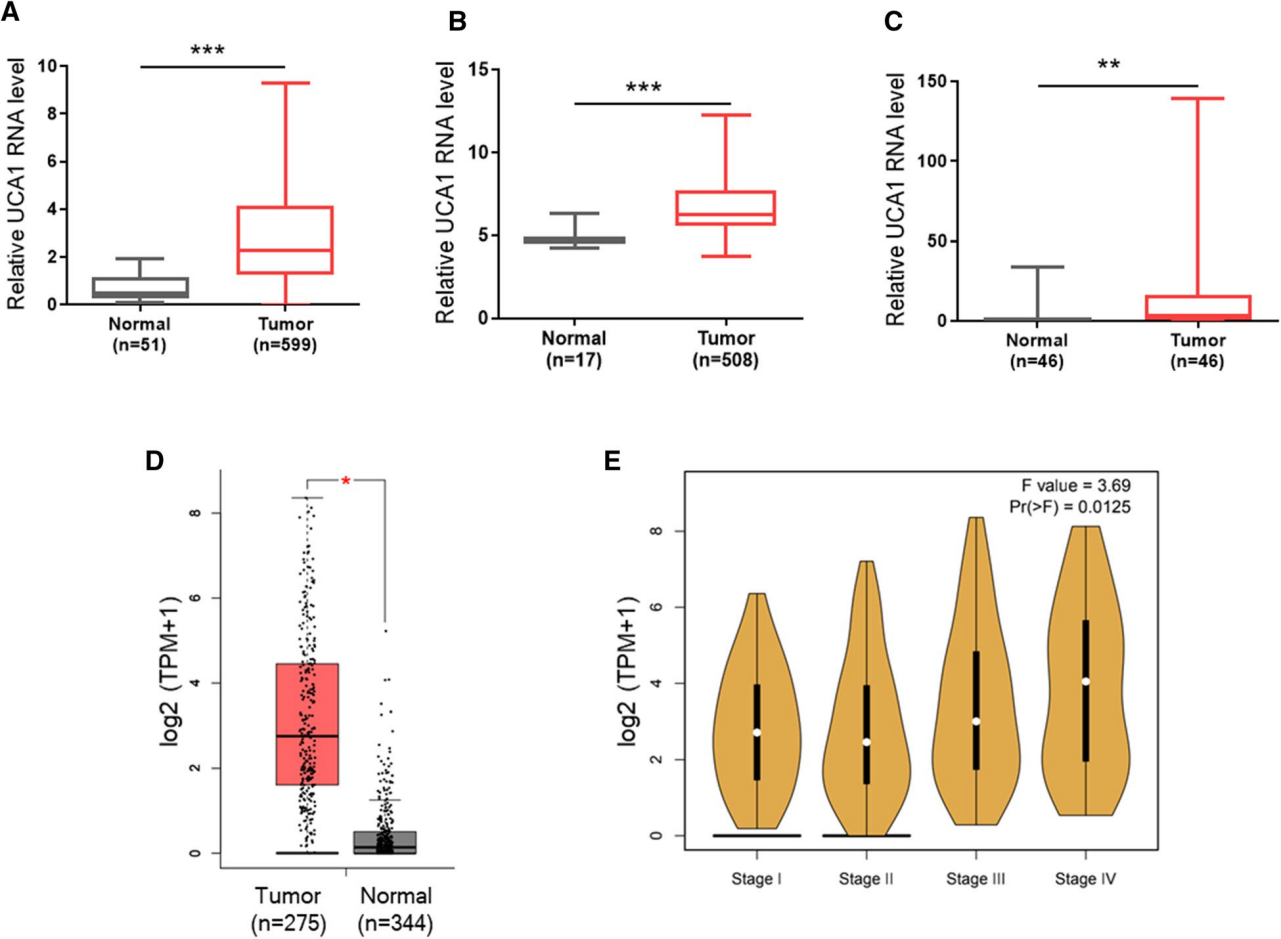
Expression of UCA1 in CRC clinical specimens. **A** Analysis of TCGA CRC cohort revealed that the expression of UCA1 is significantly higher in CRC tissue than in normal tissue. **B** UCA1 expression in 443 colon cancer samples and 19 colon mucosa tissue from GEO database (GSE39582) **C** qRT-PCR analysis of UCA1 expression in our cohort containing 46 matched CRC tissues and adjacent normal tissues. **D** GEPIA database was used to analyze the differential UCA1 expression between CRC samples and normal controls (|Log2FC| cutoff = 1; p-value cutoff = 0.05). **E** UCA1 expression was associated with the pathological stage of CRC based on GEPIA database analysis. Error bars represent SD; *P < 0.05; **P < 0.01; ***P < 0.005

To determine the expression of UCA1 in CRC, we first analyzed CRC data from TCGA. We found that the level of UCA1 was significantly higher higher in CRC (n = 599) than in normal tissues (Fig. 1A). Analysis of GEO data (GSE39582) also revealed that UCA1 was upregulated in CRC tissues (Fig. 1B). Next, we further determined the expression of UCA1 in 46 pairs of CRC tumor tissues collected from our hospital by qRT-PCR. Our data showed that UCA1 was significantly higher in CRC tissues than matched adjacent normal tissues (Fig. 1C). Finally, analysis of GEPIA database indicated that UCA1 was upregulated in CRC tissue as compared to normal tissues (Fig. 1D). Furthermore, UCA1 expression was associated with the pathological stage of CRC (Fig. 1E).

### Knockdown of UCA1 suppresses cell proliferation in vitro and tumor growth in vivo

**Fig. 2:**
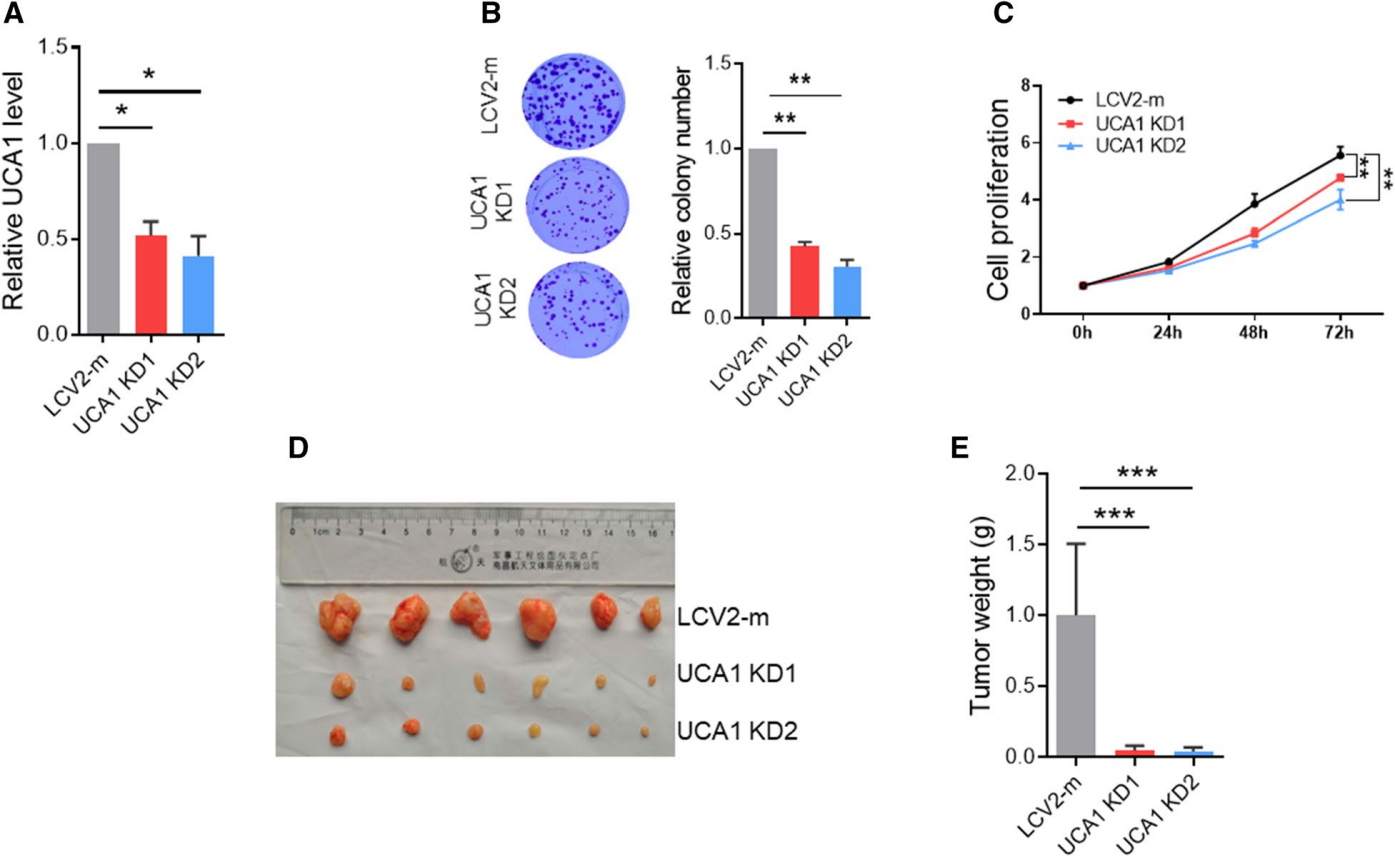
Knockdown of UCA1 suppresses cell proliferation in vitro and tumor growth in vivo. **A** The RNA level of UCA1 in HCT-116 UCA1 knockdown (KD) cells as determined by qRT-PCR. **B** UCA1 KD suppresses colony formation of HCT116 cells. Left, the representative picture of cell colonies; right, the statistical result. **C** UCA1 KD impairs cell proliferation ability of HCT116 cells as determined by CCK-8 assay. **D** and** E** UCA1 KD suppresses the tumor growth in vivo. Tumors were harvested at the end of experiment. **D** the picture of tumors; **E** the statistical analysis of tumor weight. Error bars represent SD; n = 3 for A ~ C; *P < 0.05; **P < 0.01; ***P < 0.005

To determine the function of UCA1 in CRC, we knocked out UCA1 in HCT116 cells with CRISPR/Cas9 system. Based on qRT-PCR results, it is apparent that they are partial knockout clones, i.e., knockdown (KD). For example, both UCA1 KD1 and UCA1 KD2 suppressed UCA1 expression by ~ 50% (Fig. 2A). Importantly, colony formation assay showed that UCA1 KD significantly suppressed colony forming abilities of HCT116 cells as compared with controls (Fig. 2B). CCK8 assay also showed that UCA1 KD inhibited HCT116 cells proliferation (Fig. 2C). To demonstrate the function of UCA1 in vivo, we injected HCT116 UCA1 KD or vector control cells into nude mice. The tumor volumes were significantly less and the tumor weight also much lower in UCA1 KD group than that in vector control group (Fig. 2D–E). These results suggest that UCA1 acts as an oncogene in CRC.

### UCA1 is modified by m6A

**Fig. 3:**
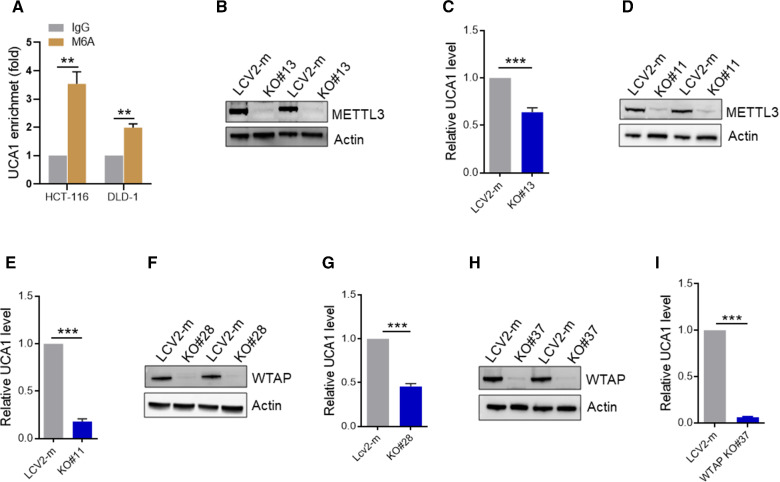
Knockout of m6A writers downregulates UCA1 expression. **A** Me-RIP and qRT-PCR assays suggest the m6A modification of UCA1 in HCT-116 and DLD-1 cells. **B** and **C** Knockout of METTL3 impairs UCA1 expression in HCT-116 cells. **B** Representative western blot result showed knockout of METTL3 in HCT-116 cells. **C** downregulation of UCA1 in HCT-116 METTL3 KO cells was detected by qRT-PCR.** D** and** E** Knockout of METTL3 impairs UCA1 expression in DLD-1 cells. **D**, Representative western blot showing knockout of METTL3 in DLD-1 cells. **E** downregulation of UCA1 in DLD-1 METTL3 KO cells was detected by qRT-PCR. **F** and **G** Knockout of WTAP suppresses UCA1 expression in HCT-116 cells. **F** Representative western blot showing knockout of METTL3 in HCT-116 cells. **G**, downregulation of UCA1 in HCT-116 WTAP KO cells was detected by qRT-PCR.** H** and **I** Knockout of WTAP suppresses UCA1 expression in DLD-1 cells. **H**, Representative western blot showing knockout of WTAP in DLD-1 cells. **I** downregulation of UCA1 in DLD-1 WTAP KO cells was detected by qRT-PCR. Error bars represent SD, n = 3, **P < 0.01, ***P < 0.005

To determine what factors contribute to upregulation of UCA1, we tested whether RNA methylation plays a role in this aspect because increasing evidence suggests that like mRNAs, lncRNAs can also be regulated by m6A modification [17, 23]. Thus, we performed RNA immunoprecipitation assay with m6A antibody in HCT116 and DLD-1 cells. As expected, there was over a threefold enrichment of UCA1 over the IgG control in HCT116 (Fig. 3A); similarly, there was about a twofold enrichment of UCA1 over the IgG control in DLD-1 cells (Fig. 3A), suggesting the possible involvement of m6A in regulation of UCA1.

To further determine role of m6A modification in UCA1 expression, we knocked out two key m6A writers (METTL3 and WTAP, respectively) with CRISPR/Cas9 in colorectal cell lines HCT116 and DLD-1. The knockout (KO) clones were identified by Western blot. Shown in Fig. 3B is METTL3 KO in HCT116 cells. Of great interest, we found that METTL3 KO significantly downregulated UCA1 expression (P < 0.005) (Fig. 3C). We also found that METTL3 KO significantly downregulated UCA1 expression in DLD1 cells (P < 0.005) (Fig. 3D–E). Like METTL3, WTAP KO also downregulated UCA1 expression in both cell lines (Fig. 3F–I).

**Fig. 4:**
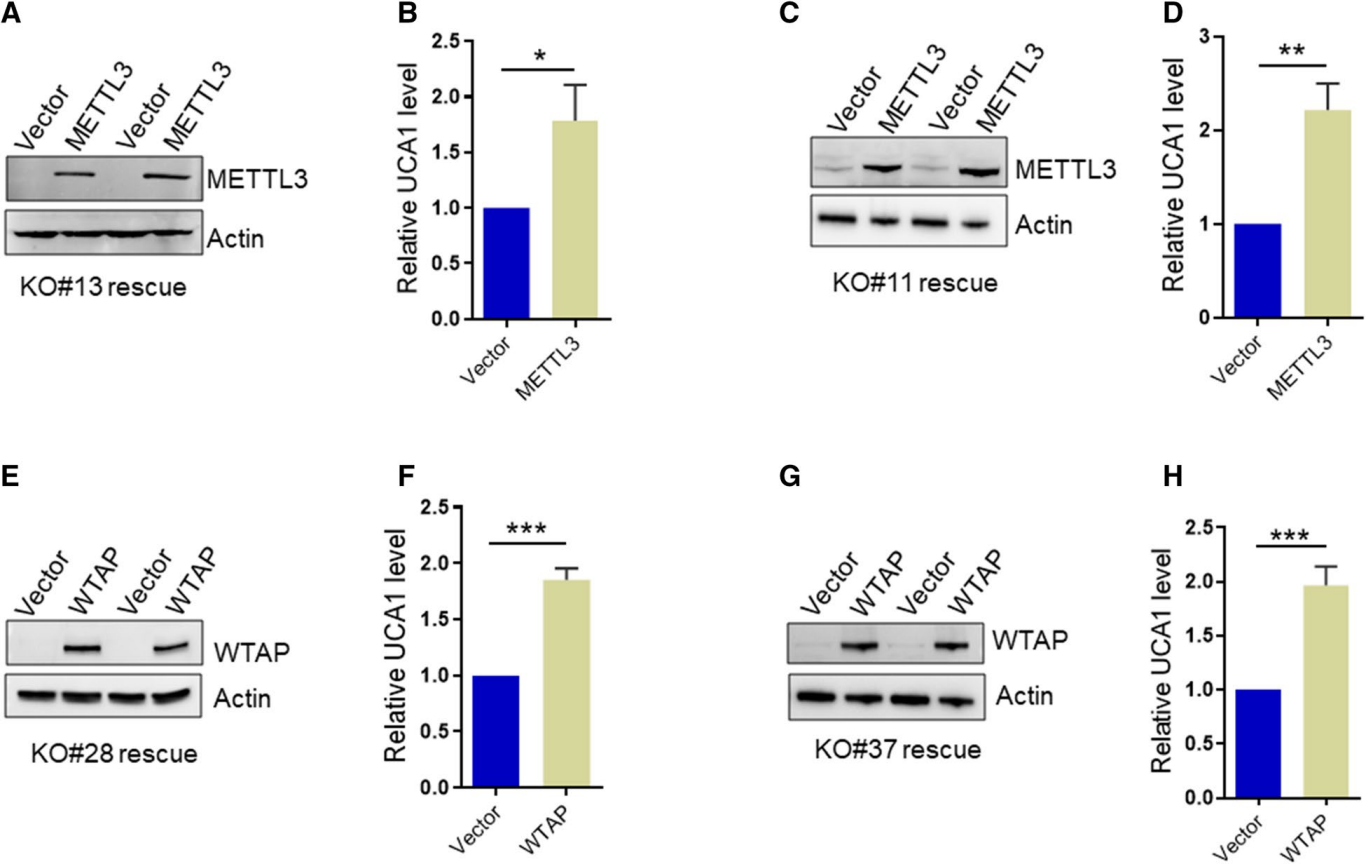
Re-expression of m6A writer restores the expression of UCA1 in KO cells. **A**–**D** Re-expression of METTL3 in KO cells restores UCA1 RNA level. **A** and **C** the representative western blot showing re-expression of METTL3 in HCT-116 KO clone#13 and DLD-1 KO clone#11. **B** and **D** the RNA level of UCA1 was detected by qRT-PCR. **E**–**H** Re-expression of WTAP in KO cells rescues UCA1 RNA level. **E** and **G** the representative western blot showing re-expression of WTAP in HCT-116 KO clone#28 and DLD-1 KO clone#37. **F** and **H** the RNA level of UCA1 was detected by qRT-PCR. Error bars represent SD, n = 3, *P < 0.05, **P < 0.01, ***P < 0.005

To further confirm the METTL3-mediated UCA1 expression, we performed rescue experiments, i.e., re-expression of METTL3 in the KO HCT116 and DLD-1 cells (Fig. 4A & C). As expected, re-expression of METTL3 restored UCA1 expression (Fig. 4B & D). Similarly, re-expression of WTAP in the WTAP KO cells increased UCA1 expression level in HCT116 and DLD1 cells (Fig. 4E–H). Together, these results suggest that UCA1 is positively regulated by m6A writers.

### Knockout of METTL3 and WTAP suppresses cell proliferation

**Fig. 5:**
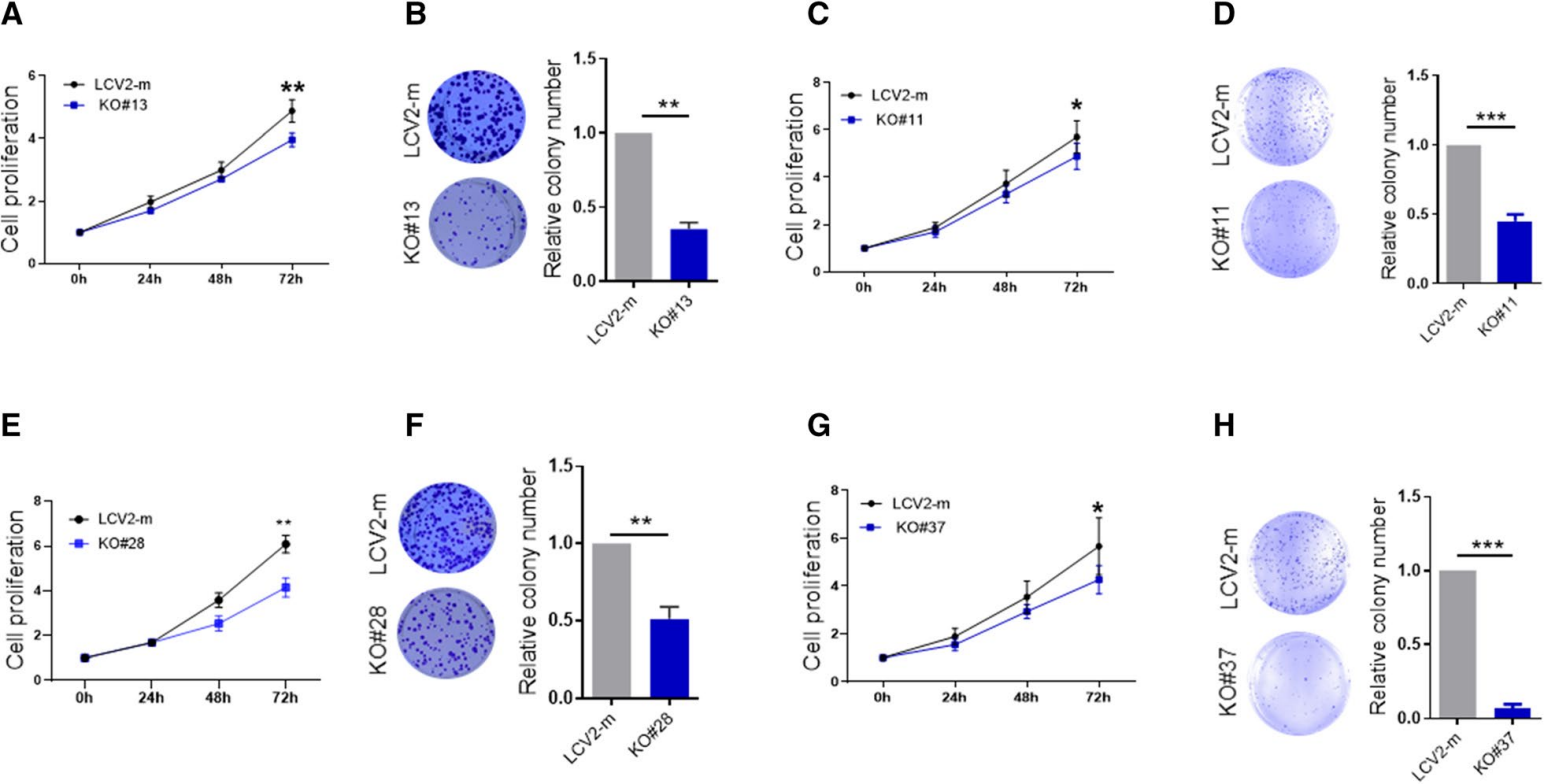
Knockout of METTL3 and WTAP impairs proliferation and survival of CRC cells. **A** METTL3 KO suppresses cell proliferation of HCT-116 cells, as determined by CCK-8 assay. **B** METTL3 KO inhibits colony formation of HCT-116 cells. Left, the representative picture of cell colonies; right, the statistical result. **C** and **D** Cell proliferation and colony formation assay in METTL3 KO DLD-1 cells. **E** KO of WTAP suppressed cell proliferation of HCT-116 cells. Cell proliferation was determined by CCK-8 assay. **F** WTAP KO inhibits colony formation of HCT-116 cells. Left, the representative picture of cell colonies; right, the statistical result. **G** and **H** Cell proliferation and colony formation assay in WTAP KO DLD-1 cells. Error bars represent SD, n = 3, *P < 0.05, **P < 0.01, ***P < 0.005

Since we have shown that METTL3 and WTAP positively regulate UCA1 expression, we set up to explore the biological function of METTL3 and WTAP in CRC. METTL3 KO inhibited cell proliferation abilities in HCT116 cells (Fig. 5A). A similar effect was also seen in DLD-1 cells (Fig. 5C). Colony formation assays revealed that METTL3 KO significantly suppressed cell survival both HCT116 and DLD-1 cells (Fig. 5B & D). In addition, WATP KO inhibited colony forming abilities and cell proliferation of HCT116 and DLD1 cells (Fig. 5E–H). There results suggest that m6A writers are involved in regulating CRC cell proliferation at least in part through regulation of UCA1 expression.

### IGF2BP2 serves as a reader for m6A-modified UCA1

**Fig. 6:**
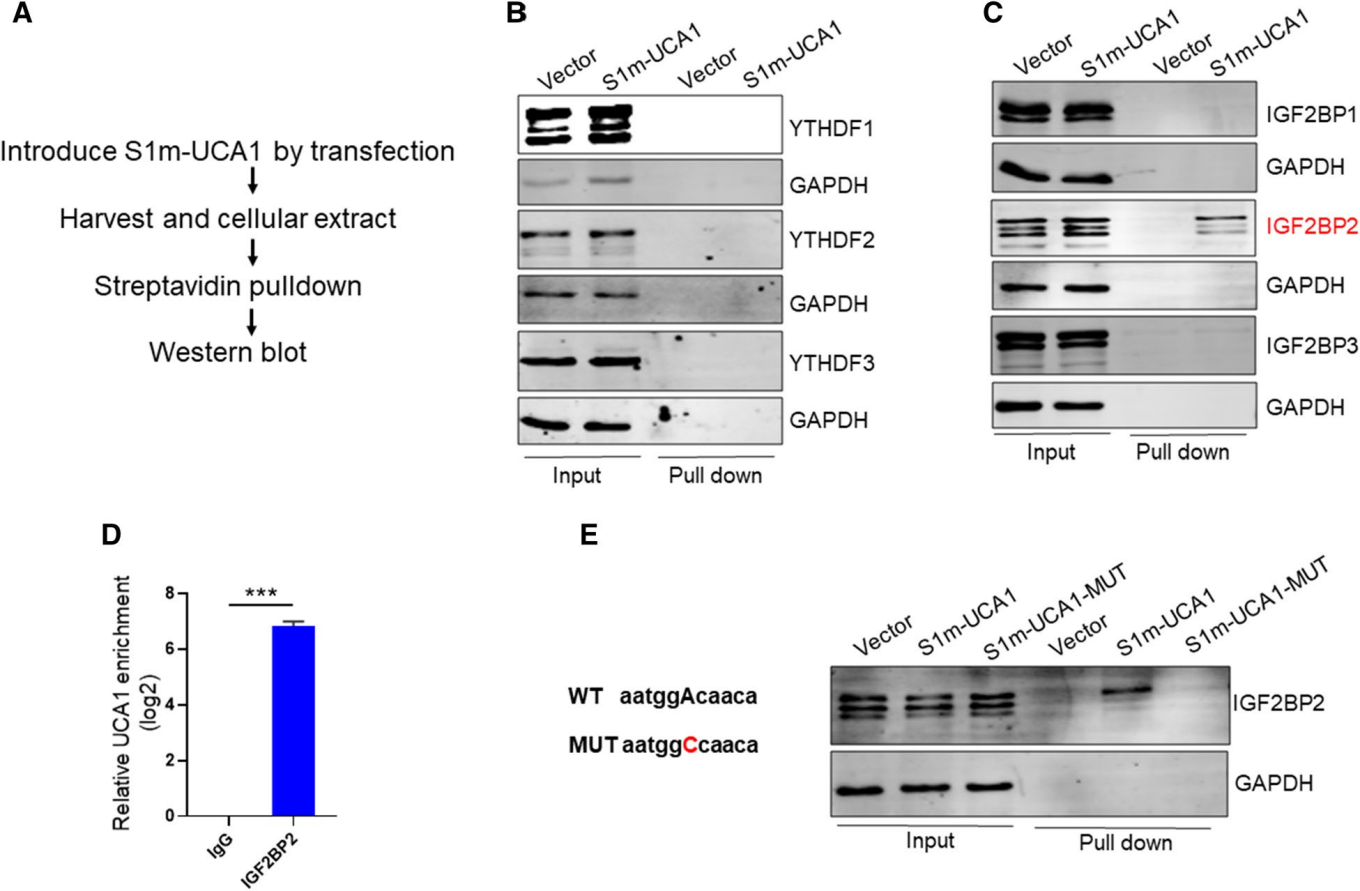
IGF2BP2 serves as a reader for m6A modified UCA1. **A** The workflow of in vivo S1m-tagging RNA pulldown assay. **B** Detection of the YTHDF1, YTHDF2 and YTHDF3 by western blot, after in vivo RNA pulldown.** C** Detection of the IGF2BP1, IGF2BP2 and IGF2BP3 by western blot, after in vivo RNA pulldown. **D** The interaction between UCA1 and IGF2BP2 was confirmed by RIP assay. Error bars represent SD, n = 3, ***P < 0.005. **E** Mutation on m6A motif of UCA1 impairs the interaction of IGF2BP2 with UCA1. Left, the schematic of the A to C mutation on UCA1 m6A motif; right, the representative western blot showing that the mutant UCA1revealed little interaction with IGF2BP2 and UCA1, in contrast to WT UCA1

The m6A readers recognize m6A and affect the fate of the methylated RNA. There were two major m6A “readers” families, YTH family and IGF2BP family. To determine which m6A reader is involved in UCA1 m6A, we performed in vivo RNA pulldown assay to identify UCA1 m6A readers using the procedure outlined in Fig. 6A. In this case, we first transfected the cells with S1m tagged UCA1 and harvested the cells one day after transfection. The cellular extract was subject to streptavidin beads because S1m functions as biotin that can interact with streptavidin [24], which was derived from a streptavidin-binding aptamer termed S1 [25, 26]. After precipitation the samples were separated in SDS-PAGE, followed by probing with antibodies against the two families of readers, i.e., IGF2BP and YTH family. This in vivo pulldown assay identified IGF2BP2 as a potential reader for m6A-modified UCA1; in contrast, there was no interaction for the other members (Fig. 6B & C). To further confirm this result, we performed the RNA immunoprecipitation (RIP) assays with IGF2PB2 antibody. There was over a 100-fold enrichment of UCA1 by IGF2PB2 antibody over IgG control (Fig. 6D). Bioinformatics analysis identified a potential m6A motif at nt 1038. Thus, we mutated the “A” to “C” (Fig. 6E, left). S1m precipitation showed IGF2BP2 bound to WT UCA1, but not the mutant UCA1 (Fig. 6E, right). Together, these findings suggest that m6A-modified UCA1 is recognized by IGF2BP2.

**Fig. 7:**
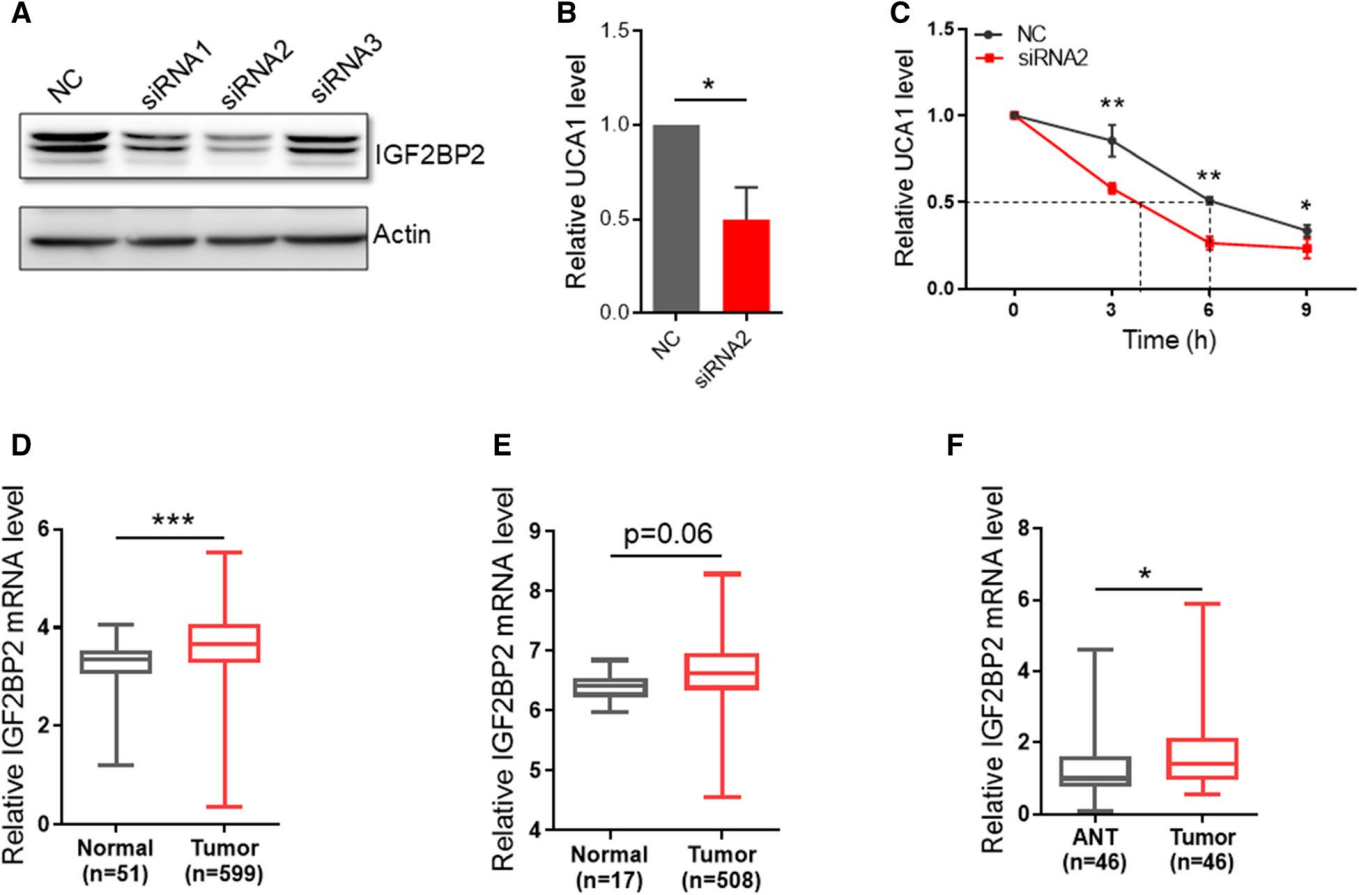
The recognition by IGF2BP2 enhances UCA1 RNA stability.** A** silencing of IGF2BP2 suppressed IGF2BP2 RNA expression, as determined RNA expression, as determined by western blot. **B** IGF2BP2 siRNA2 suppresses UCA1 RNA level, as determined by qRT-PCR. **C** IGF2BP2 siRNA2 decreases UCA1 RNA stability. HCT-116 cells were transfected with IGF2BP2 siRNA2 or NC siRNA. Two days after transfection, the cells were treated with actinomycin D (2 μg/ml) for indicated times. UCA1 RNA stability was determined by qRT-PCR. **D** Analysis of TCGA database suggests that IGF2BP2 mRNA level is higher in CRC samples than in normal tissue. **E** Analysis of GEO database (GSE39582) indicates the differential expression of IGF2BP2 in CRC tissue and normal tissue. **F** qRT-PCR analysis of IGF2BP2 expression in our cohort of 46 matched CRC tumor tissues and adjacent normal tissues; Error bars represent SD, n = 3 (B&C), *P < 0.05, **P < 0.01, ***P < 0.005

To determine the consequences of interaction between UCA1 and IGF2BP2, we suppressed expression of IGF2BP2 by IGF2BP2 siRNAs. As shown in Fig. 7A, UCA1 expression was significantly decreased by IGF2BP2 siRNAs, particularly siRNA2. Of great interest, IGF2BP2 siRNA was able to suppress UCA1 expression (Fig. 7B). To address how IGF2BP2 regulates UCA1 expression, we tested the UCA1 RNA stability because IGF2BP2 can serve as an RNA stabilizer [27]. In this case, HCT116 cells were transfected with IGF2BP2 siRNA2 or negative control siRNA and then treated with actinomycin D, and total RNA extracted at different time points. As shown in Fig. 7C, the UCA1 decay in IGF2BP2 siRNA2 cells was faster than that in control cells in HCT116 cells.

We then reasoned that as a UCA1 regulator, IGF2BP2 may also play a role in CRC. Thus, we analyzed IGF2BP2 expression in TCGA CRC data, and the expression of IGF2BP2 was significantly increased in CRC as compared to normal tissues (Fig. 7D). Analysis of GSE3958 dataset also showed upregulation of IGF2BP2 in CRC although it was not significant (Fig. 7E). Finally, analysis of our 46 pairs of CRC tumor tissues qRT-PCR showed that IGF2BP2 was higher in CRC tissues than in matched normal tissues (Fig. 7F).

## Discussion

**Fig. 8:**
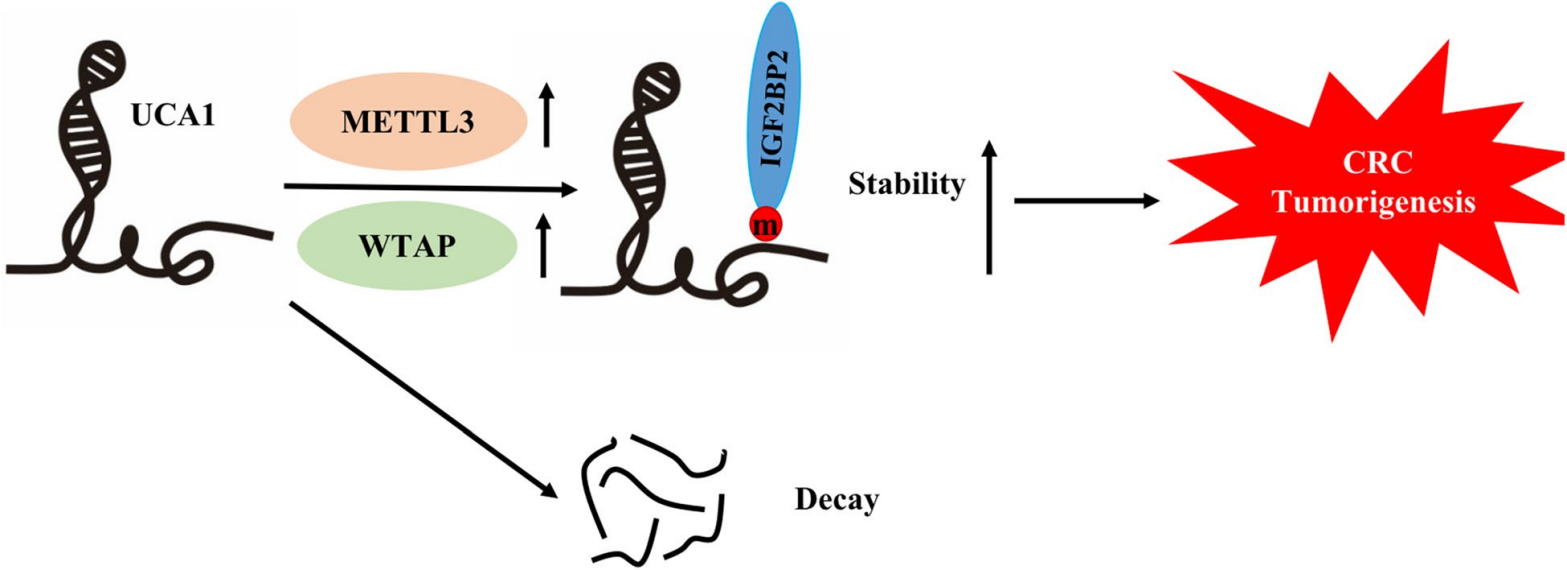
A schematic model of present work. IGF2BP2 stablizes m6A-modified UCA1 to promote CRC progression. See text for explanation

Although UCA1 was initially identified in human bladder cancer cell line, subsequent studies revealed that it is overexpressed in a wide range of human cancers, including CRC [28, 29]. Extensive studies regarding the role of UCA1 in cancers have uncovered that UCA1 promotes cancer development by various mechanisms. For example, UCA1 promotes gastric cancer cell proliferation by activation of AKT via recruiting EZH2 [30]. A recent study reported that highly expressed UCA1 confers renal cancer cells malignant phenotype by acting as a ceRNA [31]. In CRC, UCA1 has been implicated into not only in malignant phenotype but in chemo-resistance by interacting with cancer-promoting signaling pathways, through sponging tumor suppressor miRNAs [32–34]. One group has reported UCA1-SP1/SP3 may form a positive feedback loop in CRC to confer its overexpression in CRC [35]. Thus, it is commonly accepted that UCA1 plays important role in CRC progression, however, little is known regarding the underlying mechanisms that maintain its high level in CRC. Our study indicates that m6A modification plays a regulatory role in UCA1 expression in CRC. Specifically, the m6A writers WTAP and METTL3, and the m6A reader IGF2BP2 positively regulate its expression (Fig. 8). Consistent with the finding of upregulation of UCA1 in CRC, METTL3, WTAP and IGF2BP2 are also upregulated, supporting the clinical importance of this regulatory system.

N^6^-methyladenosine (m^6^A) modification, the most frequent chemical modification in eukaryotic RNA, is involved in many biological processes including cancer progression [36, 37]. Emerging studies highlighte that m6A modulates gene expression, thereby regulating tumor development aspects from cancer cell growth, metastasis, self-renewal of cancer stem cells, and apoptosis [38, 39].

M6A modification is a dynamic process and three key players, i.e., writers, erasers and readers, fine tune the dynamic balance of m6A level in cells [40]. Writers are methyltransferases that directly add methyl group to N6 position of adenosine. They usually have specific consensus motifs for RNA substrates. At least three writers have been identified and they are METTL3, METTL14 and WTAP. On the other hand, readers consist of large numbers of RNA binding proteins, including YTH protein family and IGF2BP protein family. We focus on regulation of UCA1 by METTL3 and WTAP, and IGF2BP2 in this study.

In this regard, we show that UCA1 expression can be regulated by m6A modification in CRC. Three lines of evidence support the regulatory role of the m6A system in UCA1 expression. (1) KO of METTL3 and WTAP causes a reduction of UCA1 level; (2) re-expression of METTL3 or WTAP in the corresponding KO cells increases UCA1 expression; (3) similarly, IGF2BP2 can also positively regulate UCA1 level. In addition, there is a positive correlation between UCA1 and METTL3, UCA1 and WTAP in the CRC clinical specimens. Given that UCA1 plays an oncogenic role in a variety of cancer, these findings support the importance of m6A-mediated UCA1 expression.

As critical components of the m6A system, both METTL3 and WTAP have been shown to play an important role in various types of cancers. For example, METTL3 has been reported as an important oncogene in many tumor types, such as liver cancer, breast cancer and CRC [41–43]. It has been shown that WTAP is overexpressed in variety of cancers [44–46]. Our study suggest that UCA1 is an important substrate for m6A, upregulation of UCA1 by m6A-modification promotes UCA1-mediated cancer cell proliferation and survival.

IGF2BP2 is an important RNA binding protein, which regulates RNA location, stability and translation. Moreover, accumulating evidence supports the role of IGF2BP2 in cancer. For example, the up-regulated IGF2BP2 promotes pancreatic cancer cell proliferation by activating the PI3K/Akt signaling pathway [47]. Our recent study demonstrated that IGF2BP2 stabilizes DANCR RNA to confer the stemness of pancreatic cancer cells [48]. Importantly, IGF2BP2 is significantly increased in CRC tissues and high level of IGF2BP2 is correlated with shorter overall survival [43, 49]. In present study, we demonstrated that upregulation of IGF2BP2 in CRC tissues as compared to normal tissue, which is also supported by the analysis of TCGA and GEO datasets. Of interest, IGF2BP2 has been shown to serve as a novel m6A reader, and is involved in tumorigenesis. For example, we have recently shown that IGF2BP2 recognizes the m6A modified DANCR and promotes pancreatic cancer pathogenesis [17]. The present study suggests that m6A modified UCA1 can also be recognized by IGF2BP2 and this recognition increases the UCA1 level by enhancing its stability. Apparently, UCA1 and DANCR might not be the only lncRNA targets for IGF2BP2 and we expect that more lncRNA targets for IGF2BP2 will be identified in near future.

In summary, we demonstrate that m6A plays an important role in regulation of UCA1. In this m6A system, m6A-modified UCA1 is recognized by IGF2BP2, which subsequently stabilizes UCA1. Clinically, like UCA1, METTL3, WTAP and IGF2BP2 are also upregulated in CRC tissues. Therefore, further dissection of this regulatory system will provide new insight into UCA1-mediated tumorigenesis in CRC.

## Supplementary Information

**Additional file 1. Table 1:** The list of primers used in this study.

## Data Availability

The data used to support the findings of this study are included within the article and the materials will be available once request.
