# Supplementary material for: Stabilization of UCA1 by N6-methyladenosine RNA methylation modification promotes colorectal cancer progression
Source: Cancer Cell Int. 2021 Nov 22;21:616. doi: 10.1186/s12935-021-02288-x (PMC8609784; doi:10.1186/s12935-021-02288-x)
Supplement: Supplementary file 1 — Additional file 1. Table 1: The list of primers used in this study. [file 12935_2021_2288_MOESM1_ESM.docx]

UCA1-RT-5.1A CCAATTTCAAATCGGATCTC

UCA1-RT-3.1A TTGGTGTGCTATAAATGAC

WTAP-RT-5.1 GCGACTAGCAACCAAGGAAC

WTAP-RT-3.1 CATTTTGGGCTTGTTCCAGT

METTL3-RT-5.1 GAGTGCATGAAAGCCAGTGA

METTL3-RT-3.1 ACTGGAATCACCTCCGACAC

IGF2BP2-RT 5.1 TATATCGGAAACCTCAGCGAGA

IGF2BP2-RT 3.1 CCGCAGCGGGAAATCAATCT

β-Actin 5.1 TGGATCAGCAAGCAGGAGTA

β-Actin 3.1 TCGGCCACATTGTGAACTTT

UCA1-T1a GTGCATGGTGGAGAGATGAT

UCA1-T1b TTCTGGAATGGTGAACCCAA

Mettl3-T1a Ggacacgtggagctctatcc

Mettl3-T1b gaagcaggactcggggcact

WTAP-T3a actttcactggaagaaaata

WTAP-T3b CAAGAGATGAGTTAATTCTA

Control siRNA (NC) UUCUCCGAACGUGUCACGUTT

IGF2BP2-siRNA1 GCUGUUAACCAACAAGCCATT

IGF2BP2-siRNA2 GCGAAAGGAUGGUCAUCAUTT

IGF2BP2-siRNA3 ACAGGACUGUCCGUGCUAUTT
